# Prevalence and risk factors of chronic kidney disease among Palestinian type 2 diabetic patients: a cross-sectional study

**DOI:** 10.1186/s12882-020-02138-4

**Published:** 2020-11-16

**Authors:** Zaher Nazzal, Zakaria Hamdan, Dunia Masri, Oday Abu-Kaf, Mohammad Hamad

**Affiliations:** 1 Department of Family and Community Medicine, Faculty of Medicine and Health Sciences, An-Najah National University, Box 7, 707, Nablus, Palestine; 2 Department of Internal Medicine, Faculty of Medicine and Health Sciences, An-Najah National University, Nablus, Palestine; 3 Department of Nephrology, An-Najah National University Hospital, Nablus, Palestine; 4 Faculty of Medicine and Health Sciences, An-Najah National University, Nablus, Palestine

**Keywords:** Prevalence, Diabetes mellitus type two, Primary health care, Palestine, Renal insufficiency

## Abstract

**Background:**

Chronic kidney disease (CKD) is a global public health concern and diabetes is one of the main risk factors for its occurrence and progression. The aim of this research is to determine the prevalence of chronic kidney disease in a cross-sectional population of patients with type 2 diabetes in primary health centers in North West Bank.

**Methods:**

Patient data including patient characteristics, creatinine level, blood pressure, HbA1c, and hypertension and period of diabetes were obtained from primary health care centers. The eGFR has been determined using the CKD-EPI equation. CKD was staged according to the 2012 Kidney Disease Improving Global Outcomes Framework (KDIGO) guideline. Both univariable and multivariable statistical analysis was conducted using SPSS.

**Results:**

The prevalence of chronic kidney disease among diabetic adults in North West Bank was found to be 23.6% (95% CI: 19.4–28.1%) divided as follows: 19.7% had stage 3 CKD, 2.6% had stage 4 CKD and 1.3% had stage 5 CKD. In multivariable logistic regression, CKD was significantly associated with Age ≥ 60 years [adjusted OR: 3.2, 95% CI: 1.8–5.9], hypertension [adjusted OR: 5.7, 95% CI: 2.2–15.2], and smoking [adjusted OR: 2.3, 95% CI: 1.3–4.2].

**Conclusions:**

CKD is very prevalent among diabetic adults in Palestine. Co-morbid hypertension, smoking and older age has been shown to increase the risk of developing CKD. Extensive screening for diabetic patients to diagnose CKD at an early stage and to follow more aggressive treatment methods for diabetes as well as other important risk factors, especially hypertension and smoking, is recommended.

## Background

Non-communicable diseases (NCDs), such as diabetes and kidney disease, are the leading cause of death and morbidity worldwide [1]. Diabetes Mellitus (DM) is known as the fastest-growing chronic disease in the world. Worldwide, one in every 11 adults has DM, 90% of whom have type 2 diabetes mellitus (T2DM). This number has grown tremendously over the last three decades due to rising rates of sedentary lifestyle, unhealthy diet, smoking and alcohol consumption [2]. Unfortunately, all Arab countries in the Middle East and North African regions are burdened with the second highest prevalence of diabetes [3]. The prevalence of T2DM among Palestinians living in the West Bank was 15.3% in 2010 and is expected to rise to 20.8 and 23.4% in 2020 and 2030 [4].

Chronic DM hyperglycemia is known to be one of the main causes of Chronic Kidney Disease (CKD) in addition to hypertension. It is defined as renal structure or function abnormalities, present for > 3 months, with health implications. CKD is graded on the basis of origin, category GFR and category Albuminuria (CGA) [5].

CKD is a major public health problem, both for its high cost of morbidity and treatment. The effects of CKD include not only progression to renal failure, but also decreased complications of renal function and increased risk of cardiovascular disease and overall mortality from all causes [6]. Global Burden of Disease study for 2015 reported that 1,2 million people died as a result of renal failure in 2015, a rise of 32% since 2005 [7]. Unfortunately, the mild form of kidney disease is generally under-diagnosed and under-treated [8], especially in the Arab world [9], which leads to the loss of prevention opportunities.

Early diagnosis and proper management prevent or delay many complications of CKD. In addition, early detection of kidney disease can delay or avoid deterioration of kidney function through affordable interventions, some of which are on the WHO’s so-called best-buys list for non-communicable disease management [10].

There is a shortage of data in the Middle East on the prevalence of CKD among diabetic patients. However, the prevalence of CKD among the general population was 6.8% in Jordan, 5.7% in Saudi Arabia [11] and 14.9% in Iran [12].

Common risk factors, including increased duration of diabetes, hypertension, impaired metabolic control, smoking, obesity and hyperlipidemia, have been suggested to raise the risk of diabetic complications. Low blood pressure (BP) and glycemic control have been shown to cause more kidney damage and reduced renal function. Similarly, certain context variables have been shown to be positively linked to CKD, such as age, smoking, and BMI [13–15].

In 2008, a study of diabetic hypertensive patients in North West Bank reported that 35.5% of patients with DM and HTN had decreased renal function, which is significantly correlated with age, duration of DM and number of chronic diseases [16].

GFR is the best indicator of kidney function, taking into account age, race and sex. The two most popular methods for calculating GFR are currently creatinine clearance and the approximate GFR (eGFR) [17]. Formula-derived eGFR results have become widely used in clinical practice and have been recommended by the UK National Renal Services Framework for the annual assessment of all patients with diabetes [18].

The identification of risk factors for deterioration of renal function is important for the development of preventive measures for the management of diabetic patients and for the prevention of their complications. The objective of this study is to estimate the prevalence of CKD among Palestinian diabetic patients and to determine the associated risk factors.

## Methods

### Study design and population

This is a cross-sectional study of Palestinian diabetic adult patients in the West Bank. In Palestine, all patients diagnosed with DM are referred to the Primary Health Care (PHC) directorates in their cities, where they receive diabetes care, treatment and follow-up on a regular basis. Patients with T2DM and > 18 years of age were included in the study. However, type 1 diabetes mellitus, gestational diabetes mellitus, pregnant women and patients who did not have at least 2 serum creatinine readings of at least 3 months apart were excluded from the study.

The following sample size formula was used to calculate the sample size [Necessary Sample Size = Z^2^ * expected CKD prevalence *(1- expected CKD prevalence) / (margin of error)^2^] [19]. A sample size of 385 patients was calculated assuming a 95% confidence level, 0.5 expected CKD prevalence (the maximum variability) and a margin of error ± 5%. Patients were picked at random when they visited their PHC clinics. Data were obtained from personal interviews and electronic patient records between September 2018 and December 2018.

Serial serum creatinine data was obtained and eGFR was calculated using the Chronic Kidney Disease Epidemiology Collaboration formula based on serum creatinine (CKD-EPICr). CKD was described as having reduced eGFR (< 60 ml / min per 1.73 m2) for 3 months. The CKD stage was specified in accordance with the National Kidney Foundation Guideline [5], in which stage 1, 2, 3, 4 and 5 eGFRs were ≥ 90, 60 to 90, 30 to 59, 15 to 29 and < 15 ml / min per 1,73 m2 or starting dialysis therapy, respectively.

The prevalence of CKD was determined using the National Kidney Foundation Kidney Disease Outcomes Quality Initiative Classification of CKD based on eGFR alone. Albuminuria indicates stage 1 and stage 2 CKD, however, was absent in most enrolled patients and was therefore not considered to assess the level of CKD.

Participants were deemed diabetics if they had already been diagnosed with T2DM or were taking insulin or oral hypoglycemic agents. Patients were described as hypertensive if they were previously diagnosed with hypertension (HTN) or antihypertensive drugs. Obesity has been classified as BMI ≥ 30.

### Measures

Blood pressure readings have been obtained by a qualified nurse using an electronic sphygmomanometer. The height and weight were taken at the time of the interview and used to measure the BMI. Creatinine readings were collected from medical records using at least 2 readings of at least 3 months apart and the CKD-Epi formula was used to measure eGFR. Patients with eGFR < 60 mL/min/1.73 m2 in two consecutive occasions at least 3 months apart were considered to have CKD. The last available of HbA1c reading was used.

All subjects involved in the study were invited to participate on a voluntary basis after the research purpose, risk and advantage of participation were clarified. We have maintained the privacy and confidentiality of those who have agreed to participate and have been asked to sign informed consent. The approval of the Al-Najah National University Institutional Review Board (IRB), the Scientific Committee and the Palestinian Ministry of Health was received.

### Statistical analysis

Statistical analysis was performed using version 20 of the SPSS. Categorical data were expressed as number (percentage) and continuous data as means ± standard deviation (SD) or median and range, as applicable. Differences in patient’s characteristics and risk factors for CKD were analyzed using the chi-square test and unpaired student’s t-test (two-tailed), as necessary. Statistical significance is taken at a *p* value of < 0.05. Additionally, Multivariable logistic regression was performed to estimate the unique relationship between the included variables and CKD status. Variable included in the model were selected based on prior knowledge of the field [20, 21]. The linearity of continuous variables with the outcome has been checkedusing the Box-Tidwell method to test for linearity where we assumed that the relationship between continuous predictors and log (log odds) is linear. We have included in the model interactions between continuous predictors and their logs. The significance value of the deviation from linearity for BMI, duration of DM, and HbA1c was > 0.05, suggesting a linear relationship between them and CKD status.

## Results

### Study population

The study recruited 386 T2DM patients from PHC clinics in North West Bank. Almost half of the participants were male (49.7%) and their average age was 60.6 ± 10.4 years. HTN was reported in 278 (75.3%) participants, with an average period of 6.78 ± 7.7 years. The mean duration of diabetes was 12.4 years (3.9 years to 20.9 years) and the HbA1c level ranged from 6.39 to 10.47% with an average of 8.4% ± 2.0. The majority of participants were obese and 30.4% were smokers. Table 1 provides clinical and background information for patients.

**Table 1:** Clinical and Background characteristics of the participants (*n* = 386)

***Characteristics***^***a***^	***Frequency*** (***Percentage)***	Mean ± SD or median [interquartile range]
***Gender***		
Female	194 (50.3%)	
Male	192 (49.7%)	
***Age***		60.7 ± 10.4
< 60 years	168 (43.5%)	
≥ 60 years	218 (56.5%)	
***Hypertension***		
Yes	278 (75.3%)	
No	91 (23.6%)	
***Smoker***		
Yes	113 (30.4%)	
No	259 (67.1%)	
***Systolic*** *BP mmHg*		137.7 ± 18.2
***Diastolic*** *BP mmHg*		77.3 ± 11.1
***Age*** *(years)*		
***BMI*** *(kg/m*^*2*^*)*		32.7 ± 5.8
***HbA1c%***		8.4 ± 1.6
***Estimated GFR*** *(ml/min/1.73 m^2)*		75.3 ± 24
***DM Duration*** *(years)*		11.0 [6–18]
***HTN Duration*** *(years)*		7 [3–13]

^a^Continuous characteristics are presented as Mean ± SD and median [interquartile range]

### Frequency of Chronic kidney disease

**Fig. 1:**
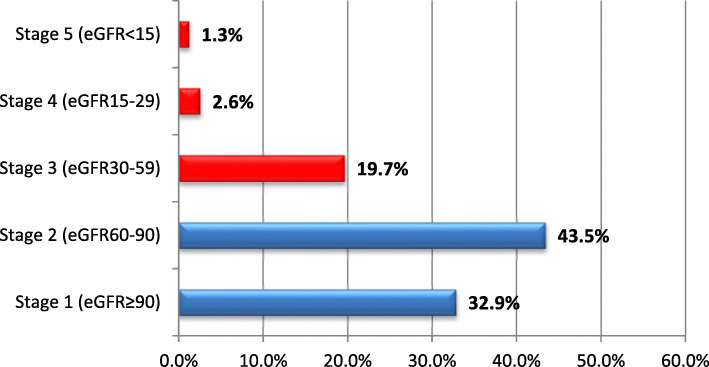
Frequencies of CKD stages based on eGFR (*n* = 386)

Using the CKD-EPI equation, the eGFR mean (±SD) for all participants was 75.3 ± 24. Estimated GFR ≥60 ml/min/1.73 m2 was reported among 295 (76.4%) diabetes patients. CKD Stages 3, 4, and 5 were present in 19.7, 2.6, and 1.3% of the participants, respectively. In total, the prevalence of CKD among T2DM patients was 23.6% (95% CI: 19.4–28.1%) (Fig. 1).

The Kruskal Wallis H test shows that there was a statistically significant difference in age, SBP, duration of HTN, and duration of DM between different CKD stages (*p* < 0.001) (Table 2).

**Table 2:** Clinical and background variables of each eGFR category

eGFR Stage (n)	5: GFR ≥ 90 (*n* = 127)	4: GFR = 60–89 (*n* = 168)	3: GFR = 30–59 (*n* = 76)	2: GFR = 15–29(*n* = 10)	1: GFR < 15 (*n* = 5)	*P* value^*^
**Age** (years)	53.1 ± 9.4	63.2 ± 8.9	67.2 ± 7.3	62.1 ± 9.8	65 ± 12.5	< 0.001
**Systolic BP** (mmHg)	133 ± 16.5	139.3 ± 17.7	139.2 ± 19.9	153 ± 15.6	154 ± 22	< 0.001
**Diastolic BP** (mmHg)	77.7 ± 11.4	76.5 ± 11.2	77.5 ± 10.5	80.1 ± 14	81.4 ± 6.8	0.883
**Hypertension Duration** (years)	6.5 ± 5	9.0 ± 7.4	11.0 ± 9	11.6 ± 13.3	12.6 ± 6.8	0.013
**HbA1c** (%)	8.58 ± 2	8.38 ± 1.98	8.57 ± 2.2	7.3 ± 1.7	6.4 ± 1.14	0.057
**Diabetes Duration** (years)	9.3 ± 6.6	12.9 ± 8.4	14.9 ± 8.8	16.8 ± 13.2	21.4 ± 9.8	0.007

^*^Kruskal Wallis test, *BP* Blood Pressure, *eGFR* Estimated glomerular filtration rate

Post-hoc analysis was performed to examine differences between classes. The ages were significantly different between stage 1 and stage 2, stage 1 and stage 3, stage 1 and stage 4, and stage 2 and stage 3. For systolic BP, the difference between stage 1 and stage 2 and stage 1 and stage 4 was significant. The duration of hypertension was substantially different between stage 1 and stage 3. The duration of the DM was significantly different between stage 1 and stage 2, stage 1 and stage 3 and stage 1 and stage 2. 5.

Unvariable analysis was performed to investigate the factors associated with the development of CKD. The findings showed that CKD is significantly associated with HTN (*p* < 0.001), smoking (*p* = 0.022), age (*p* < 0.001), and length of DM (*p* < 0.001). On the other hand, there was no significant relationship between HbA1c and BMI and CKD (Table 3). Further sub-analysis was performed to determine any differences in male / female prevalence of CKD in different age groups. The findings showed a substantial increase in prevalence of CKD among age groups for both males and females (*P* < 0.001 for both).

**Table 3:** Comparison between patients with Chronic Kidney Disease and preserved GFR (*n* = 386)

	*Multivariable Results*			*Multivariable Results*	
	Chronic Kidney Disease				
***Variables***	***Yes (n = 91)***	***No (n = 295)***	P Value*	Adjusted ***P*** Value	Adjusted OR (95% CI)
	***Frequency (%)***	***Frequency (%)***			
***Gender***					
Female	43 (22%)	151 (78%)	0.550	0.81	0.44–1.5
Male†	48 (25%)	144 (75%)			
***Age***					
< 60 years†	17 (10.1%)	151 (89.9%)	< 0.001	3.2	1.8–5.9
≥ 60 years	74 (33.9%)	144 (66.1%)			
***Hypertension***					
Yes	85 (30.6%)	193 (69.4%)	< 0.001	0.001	5.7 (2.2–15.2)
No†	05 (5.5%)	86 (94.5%)			
***Smoker***					
Yes	36 (31.85%)	77 (68.14%)	0.022	0.009	2.3 (1.3–4.2)
No†	55 (21.2%)	204 (78.76%)			
***BMI*** *(kg/m*^*2*^*)*	33 ± 5.82	32.5 ± 5.84	0.508	0.556	1.6 (0.3–9.4)
***Diabetes duration***	15.46+/−9.4	11.43+/−7.96	< 0.001	0.964	1.01 (0.74–1.4)
***HbA1c***	8.31 ± 2.2	8.47 ± 2	0.527	0.195	0.1 (0.03–3.3)

*Chi-square test and Independent t-test, *HbA1c* glycosilated hemoglobin, *OR* Odds Ratio, *CI* Confidence Interval

† Reference group

Multivariable logistic model was conducted to assess risk factors of CKD. Age ≥ 60 years (adjusted OR: 3.2, 95% CI: 1.8–5.9), HTN (adjusted OR: 5.7, 95% CI: 2.2–15.2) and smoking (adjusted OR: 2.3, 95% CI: 1.3–4.2) are found to increase the odds of CKD (Table 3).

## Discussion

Worldwide, there are an estimated 200 million people with CKD who are vulnerable to the development of ESRD if no action is taken in the early stages to diagnose and treat them [22]. ESRD is the tip of the iceberg and the total number of patients with CKD is considerably higher. It is important to study the prevalence of CKD in Palestine as it helps in the early detection and thus in the prevention and control of diabetic nephropathy..

This research was, to our best knowledge, the first epidemiological study on CKD prevalence in Palestinian primary care patients. It showed that 23.6% (95%CI 19.1–28.4) of diabetic patients in the North West Bank have CKD. Sweileh et al. reported a prevalence of 35.5% of CKD in Palestine in 2008 [16]. This research was nevertheless performed among patients with diabetic hypertensions and targeted patients with hospital diabetes, explaining the difference in prevalence. In addition, it only used one creatinine read that was presumably an acute instead of a chronic kidney injury.

Results on the prevalence of CKD among diabetic patients are variable; like Finland (16.2%) [21], Southern Ethiopia (23.4%) [23] Spain (27.9%) [20]. Unfortunately, similar data from the surrounding countries is lacking. This variation on the prevalence of CKD among Diabetic patients is attributed to a difference in the definitions adopted and the characteristics of the studied populations.

In order to improve preventive and control measures, it is important to identify the risk factors associated with CKD, in particular the modifiable factors. The prevalence rate of HTN identified in this study among patients with type 2 diabetes (75%) was high. This is more than recorded in neighboring countries; Jordan (72.4%) [24], Qatar (64.5%) [25] and Saudi Arabia (53%) [26]. This relatively higher rate of HTN may be attributed to the fact that most of the diabetic patients included in the study were obese and > 60 years of age.

This study showed a significant relationship (*P* Value < 0.001) between BP and kidney damage, represented by decreased eGFR as systolic BP increases (Table 2). Diabetic patients with HTN are more likely to develop CKD than diabetic patients with normal BP. These results are consistent with the literature of different countries [15, 20, 21]. There is a significant overlap between HTN and CKD. A vicious cycle occurs where decreased kidney function causes an increase in BP and this increase can cause more kidney damage and a subsequent decrease in kidney function.

The high prevalence of HTN among our patients is worrying and should be taken into account, as several studies have documented the association between high BP and the development of ESRD. A Japanese study has shown that the risk of developing ESRD in high BP patients is 15 times higher, compared to controlled BP patients [27]. This is important and more attention should be paid to better control of BP among diabetic patients.

Almost one-third of the patients (30.4%) were smokers; with a higher proportion of them among the CKD group (39.6%). In this study, smoking was correlated with renal function progression (*P* value = 0.022). The correlation between smoking and the occurrence of CKD in diabetic patients is clearer, with most research showing a substantial relationship between these two variables. Recently, two meta-analyses indicated evidence of cigarette smoking as an independent risk factor for CKD [14, 28]. Xia et al. have reported that the risk of CKD was 1.27 (95% CI 1.19–1.35) for ever-smokers, 1.34 (95% CI 1.23–1.47) for current smokers and 1.15 (95% CI 1.08–1.23) for former smokers, all compared to never-smokers [14]. The nephrotoxic effect of smoking, which involves endothelial cell dysfunction and increased resistance to insulin regardless of the diabetic condition, may explain this correlation [14].

Age was found to be a significant risk factor (*P* value < 0.001) for CKD. The overall high prevalence of CKD among elderly people can be explained by the steady decline in GFR with normal ageing, in addition to the high rate of comorbidities among this population, particularly hypertension and diabetes [29]. It is also likely that, because of the increased involvement of this age group with the healthcare system, CKD is more readily diagnosed than other age groups. In addition, as shown in Table 2, a substantial decrease in eGFR has been recorded as age increases. These findings suggest the need for rigorous screening of diabetic patients with an elderly diabetic emphasis.

The average BMI of diabetic patients, in this study, was 32.5 kg/m^2^ (±5.8) without a significant correlation with the renal function (*P* value = 0.508). However, obesity is a main contributor to hypertension and diabetes [30]. These two factors are well known risk factors for CKD, as several studies have shown that CKD patients have a higher prevalence of general and abdominal obesity than non-CKD patients [31–33]. On the other hand, the lack of correlation in this study can be due to a limited number of sample size.

HbA1c is a recommended standard of care for the monitoring of diabetes. In this study, the mean HbA1c was 8.31%, but it was not significant to the presence of CKD (*P* value = 0.527). Growing evidence suggests that there is a correlation between the glycemic control status and renal damage. There are contradictory details on this relationship. A research in Spain showed that HbA1c levels were significantly higher among CKD diabetic patients (OR = 1.011, 95% CI 1.005–1.017, *P* < 0.001) [20]. However, other studies have shown that the CKD stages could influence the association between HbA1c and renal outcomes. A study in Taiwan, found that HbA1c > 9% in CKD stages 3–4 was associated with increased risk for ESRD. Conversely, HbA1c is not a sufficient predictor of ESRD in patients with CKD stage 5 [34]. This is presumably due to a marked drop in insulin clearance. In addition, the development of HbA1c is known to be lower in patients with CKD due to a decrease in red blood cells lifespan, as well as resistance of carbamylated hemoglobin molecules to glycosylation in a uremic molecules [35]. Other reasons may be linked to improved treatment of diabetic patients with compromised renal function; more regular exposure to endocrine and nephrological interventions.

There was no correlation between gender and CKD in this study (*P* = 0.384). The relationship between gender and CKD among diabetic patients is inconsistent in the literature. Many studies have shown female gender as a risk factor [12, 15, 21], while others have reported male gender as a risk factor [20]. This may be due to the gender distribution of risk factors, such as obesity and T2DM control status.

The key strengths of our research include the diagnosis of CKD based on eGFR on multiple measures to establish chronicity, and undertaking the study in PHC centers in which almost all diabetic patients in Palestine receive, free of charge, their preventive and curative services.

There were some limitations to this study. First, a cross-sectional, not longitudinal, analysis precludes any causal association between CKD and its risk factors. Second, due to low resources in primary care settings, there is a lack of data on albuminuria and renal biopsy that make it difficult to diagnose stage 1 CKD. Finally, the lack of full and up-to-date patient files on drugs made it difficult to determine its relationship with CKD.

## Conclusion

This study indicates a high prevalence (23.6%) of CKD among diabetic patients in Palestine. The rate is higher among hypertensive patients and increases with age. Intensive screening for diabetic patients is recommended for early detection of CKD and for more aggressive treatment of diabetes, as well as other important risk factors, especially HTN and smoking. We also recommend studying the effect of anti-diabetic and anti-hypertensive medications on the rate of renal function deterioration, as well as evaluating the mortality rate and progression to ESRD and dialysis in each eGFR category.

## Data Availability

The datasets used and analyzed during the current study are available from the corresponding author on reasonable request.

## Abbreviations

CKD: Chronic Kidney Disease
DM: Diabetes Mellitus
GFR: Glomerular Filtration Rate
HTN: Hypertension
IRB: Institutional Review Board
KDIGO: Kidney Disease Improving Global Outcomes System
T2DM: Type 2 diabetes mellitus
